# Is antiplatelet treatment effective at attenuating the progression of white matter hyperintensities?

**DOI:** 10.1371/journal.pone.0176300

**Published:** 2017-04-20

**Authors:** Cindy W. Yoon, Yoonjae Choi, Seun Jeon, Dae Hyung Lee, Byung-Nam Yoon, Hee-Kwon Park, Joung-Ho Rha

**Affiliations:** 1 Department of Neurology, Inha University School of Medicine, Incheon, Korea; 2 McGill Centre for Integrative Neuroscience, Montreal Neurological Institute, McGill University, Montreal, Canada; 3 Center for Clinical Research, Inha University School of Medicine, Incheon, Korea; Banner Alzheimer's Institute, UNITED STATES

## Abstract

**Objective:**

We performed this study to assess the effect of an antiplatelet agent on the progression of white matter hyperintensities (WMH).

**Methods:**

From August 2003 to May 2005, we consecutively enrolled patients who underwent brain magnetic resonance imaging (MRI) for health check-up purposes and showed no significant findings other than WMH of any degree. Patients were divided into two groups based on whether or not they received antiplatelet therapy. All patients had a follow-up brain MRI after 5 years and WMH volume change was measured using imaging analysis software. To minimize selection bias potentially arising from antiplatelet treatment assignment, analyses were inverse probability weighted.

**Results:**

Among the 93 patients who met the inclusion criteria, 54 patients (58.1%) were grouped as the antiplatelet group (AG), and the remaining 39 patients (41.9%) as the non-antiplatelet group (NAG). After inverse propensity weighting, all baseline characteristics were similar between the two groups, and antiplatelet treatment did not show any significant effect on the total WMH volume change (*p* = 0.957).

**Conclusion:**

Antiplatelet medication may not alter the progression of WMH.

## Introduction

White matter hyperintensities (WMH) are bilateral patchy or diffuse hyperintense lesions in the cerebral white matter on T2 weighted or fluid attenuated inversion recovery (FLAIR) images, commonly found in the elderly [1]. The overall prevalence is about 11–20% in the general middle aged population and much higher at ages over 85 existing at almost 100% [2–5].

With increasing use of magnetic resonance imaging (MRI), many clinicians are more frequently confronted with incidental detection of asymptomatic WMH. WMH are more common in patients who have cardiovascular risk factors and symptomatic cerebrovascular disease [6], and increase the risk of stroke [7–9]. For these reasons, some clinicians prescribe an antiplatelet agent for WMH. However, to the best of our knowledge, there has been no published study on the long-term effect of antiplatelet agents for WMH. We designed and performed this study to assess the effects of an antiplatelet agent on the progression of WMH.

## Methods

### Subjects

This study was a prospective single hospital-based study. Participants who visited Inha University Hospital from August 2003 to May 2005 and underwent MRI for a preventive medical check-up were enrolled in this study. Inclusion criteria were (a) age over 45 years; (b) any degree of WMH on a brain MRI; (c) no prior use of an antiplatelet agent; and (d) agreement to a follow-up MRI scan after 5 years. Patients with any history of neurological disorders including stroke, multiple sclerosis, seizure disorders, dementia, or Parkinson’s disease and those with brain lesions associated with territorial cerebral infarction, traumatic injury, tumors, infection, multiple sclerosis, vasculitis or vascular malformations were excluded. We also excluded patients who required antiplatelet medications or anticoagulation treatment because of the presence of atrial fibrillation or systemic thromboembolism. Patients with any severe medical, psychiatric, or cognitive disorders that compromise the ability of the subject to give informed consent, comply with the study protocol, and complete the study based on the judgment of the investigator were also excluded. All participants were negative for HIV. We divided the patients into two groups. One group included the patients who were prescribed an antiplatelet agent according to the attending physician’s preference, patient’s comorbidities, and the severity of WMH at the time of the initial visit, and thus was called the antiplatelet group (AG). The other group was the non-antiplatelet group (NAG) which included patients who were not prescribed any antiplatelet medication. The study protocol was reviewed and approved by the institutional review board of Inha University Hospital, and written informed consent was obtained from all participants.

### Measurement of vascular risk factors

Vascular risk factors, including hypertension (defined as receiving medication for hypertension or a blood pressure ≥ 140/90 mmHg), diabetes mellitus (defined as the use of hypoglycemic agents, fasting blood sugar ≥ 126 mg/dl, or glycosylated hemoglobin levels ≥ 6.5%), hyperlipidemia (defined as receiving cholesterol-reducing agents or an overnight fasting low-density lipoprotein ≥ 130mg/dl), and smoking habits were also analyzed.

### Image acquisition

Using the same 1.5-tesla MRI scanner (Signa, GE Medical Systems, Milwaukee, WI, USA), basal and follow up MR images were acquired from all subjects. Axial T1, T2-weighted and FLAIR images were obtained at a slice thickness of 7 mm for all subjects.

### Measurement of WMH volume

We quantified WMH volume on FLAIR images using an automated method [10]. To minimize false positive on the quantification, we firstly generated WMH candidate mask on T1-weighted images using morphological operators. T1-weighted images were co-registered to the FLAIR images using rigid transformation. The transformation matrix was applied to the mask with the nearest neighborhood interpolation. Thus, we could define the WMH candidate regions on FLAIR images. We then computed intensity threshold value based on a hidden Markov random field model and an associated expectation-maximization algorithm within the WMH candidate region [11]. The segmented WMH was carefully verified by two raters. The threshold value was fine-tuned for the optimal result if the segmentation contains false positive or false negative. Finally, we quantified the WMH volume in the native FLAIR space. The location of WMH was divided into periventricular and deep WMH according to the modified Fazekas criteria [12]. When the largest diameter of the WMH was adjacent to the ventricle, it was defined as periventricular, otherwise, the lesion was deemed to be deep WMH.

### Normalized volume of WMH

The total volume of WMH was normalized to the intracranial volume (ICV) in order to control for brain size effects (normalized volume of WMH = [total volume of WMH/ICV] X 100). ICV was defined as the total volume of gray matter, white matter, and cerebrospinal fluid. We calculated the ICV by measuring the volume of voxels within the brain mask.

### Statistical analysis

We analyzed the differences between the AG and NAG using a chi-square test or Fisher’s exact test for categorical variables, and an independent t-test or Mann–Whitney U-test for continuous variables. To minimize selection bias potentially arising from antiplatelet treatment assignment, analyses were inverse probability weighted. We calculated inverse probability weights (IPW) using covariates including age, gender, vascular risk factors (HTN, DM, hyperlipidemia and smoking), baseline total WMH volume, and time interval between baseline and follow-up MRI scans. IPW were then used to adjust for differences between the two treatment groups. A linear regression analysis was then performed to examine the effect of antiplatelet treatment on the progression of WMH. A natural logarithmic transformation of WMH volume change was performed to achieve a normal distribution. All tests were two tailed, and p < 0.05 was considered to be statistically significant. All statistical analyses were conducted using commercially available software (SPSS, version 19.0; SPSS Inc., Chicago, IL, USA).

## Results

Table 1 shows the baseline characteristics of the study patients. Among the 93 patients who met the inclusion criteria, 54 patients (58.1%) were categorized as the AG and the remaining 39 patients (41.9%) were classified in the NAG. There was no follow-up loss in both groups. We followed up with all AG patients regularly to ensure they maintained anti-platelet agent use throughout the entire 5 years. Regarding NAG patients, when the participants visited for the follow-up MRI, we checked their medication with an interview and questionnaire to ensure they were not taking an anti-platelet agent. No NAG patients initiated anti-platelet therapy in the interim period. Among 54 AG patients, 40 patients (74%) were prescribed aspirin but other antiplatelet medications (clopidogrel 22%, cilostazol 4%) were also prescribed for some patients. There were no differences between the two groups in terms of age, gender, time interval between baseline and follow-up MRI scans, and the prevalence of diabetes, hyperlipidemia and smoking. However, the prevalence of hypertension was higher in the AG than in the NAG (38.9% vs. 15.4%, *p* = 0.020). The median total WMH volume was significantly lower in the NAG than in the AG (1602.45 mm^3^ vs. 2169.64 mm^3^; *p* = 0.026), before adjustment with the use of inverse probability weighting. However, after propensity weighting, all baseline characteristics were well balanced (Table 1).

**Table 1:** Baseline characteristics of the patients.

	Before IPW			After IPW		
	NAG	AG	*p*-value	NAG	AG	*p*-value
N	39	54		92	93	
Age, mean ± SD (years)	60.9 ± 8.7	60.5 ± 6.5	0.808	61.0 ± 8.0	60.6 ± 6.6	0.726
Male, N (%)	11 (28.2)	22 (40.7)	0.274	35 (38.0)	33 (35.5)	0.878
Hypertension, N (%)	6 (15.4)	21 (38.9)	0.020	25 (27.2)	27 (29.0)	0.870
Diabetes mellitus, N (%)	3 (7.7)	9 (16.7)	0.231	8 (8.7)	12 (12.9)	0.479
Hyperlipidemia, N (%)	2 (5.1)	6 (11.1)	0.461	12 (13.0)	8 (8.6)	0.476
Smoking, N (%)	2 (5.1)	3 (5.6)	1.000	4 (4.3)	5 (5.4)	1.000
Baseline WMH						
Absolute volume, Median (IQR) (mm^3^)						
Total	1602.45 (619.52–2375.58)	2169.64 (1165.09–3874.43)	0.026	1866.94 (721.52–3020.09)	1892.68 (1114.92–3324.54)	0.783
Deep	256.36 (146.62–610.88)	421.04 (196.54–1679.78)	0.035	400.88 (171.34–987.40)	359.81 (158.08–1008.88)	0.611
Periventricular	1229.95 (420.27–1865.31)	1451.97 (880.93–2626.17)	0.053	1402.70 (586.17–2542.06)	1228.48 (843.83–1915.21)	0.952
*Normalized volume, Median (IQR)						
Total	0.129 (0.464–0.182)	0.158 (0.089–0.275)	0.036	0.136 (0.055–0.245)	0.124 (0.082–0.237)	0.915
Deep	0.020 (0.011–0.051)	0.031 (0.016–0.133)	0.041	0.029 (0.013–0.071)	0.026 (0.011–0.070)	0.680
Periventricular	0.098 (0.031–0.136)	0.107 (0.064–0.186)	0.096	0.109 (0.043–0.210)	0.094 (0.058–0.153)	0.743
†Time interval, mean ± SD (days)	1913.6 ± 181.8	1903.4 ± 158.6	0.776	1912.6 ± 180.44	1903.8 ± 157.9	0.725

IPW = inverse probability weighting; NAG = non-antiplatelet group; AG = antiplatelet group.

N = number; SD = standard deviation; WMH = white matter hyperintensity; IQR = interquartile range.

* Normalized volume of WMH = [total volume of WMH/intracranial volume] × 100.

^†^ Time interval between the baseline and follow-up MRI scans.

Both groups showed progression of WMH. The change in total WMH volume from baseline to follow up MRI scan was not significantly different between the NAG and the AG [NAG: 619.19 mm^3^ (195.46–1504.42), median (interquartile range), vs AG: 808.53 mm^3^ (356.58–2417.02) (*p* = 0.141)]. Finally, we explored the effect of antiplatelet treatment on the progression of WMH. A natural logarithmic transformation of WMH volume change was performed to achieve a normal distribution. Antiplatelet treatment did not show any significant effect on total WMH volume change in both unadjusted univariate analysis (*p* = 0.105) and IPW adjusted analysis (*p* = 0.957). The effects on deep and periventricular WMH volume changes were analyzed separately but there were also no significant protective effects of antiplatelet agents (Table 2).

**Table 2:** The effect of antiplatelet treatment on the progression of white matter hyperintensities (WMH).

^†^WMH volume change	Univariate unadjusted		IPW adjusted	
	B (std. error)	*p*-value	B (std. error)	*p*-value
Total	-0.538 (0.329)	0.105	-0.012 (0.225)	0.957
Deep	-0.671 (0.382)	0.082	-0.108 (0.267)	0.686
Periventricular	-0.498 (0.354)	0.163	-0.041 (0.237)	0.864

IPW = inverse probability weighting; B = beta; std. error = standard error.

^†^A natural logarithmic transformation of WMH volume change was performed to achieve a normal distribution.

## Discussion

WMH are relatively common in the elderly. Previous studies have shown the incidence of WMH increases with age, existing at nearly 100% in the population over 85 [2–5], and the progression of WMH over time is substantial in a significant proportion of elderly people [13]. WMH prevalence is greater in individuals with higher levels of and longer exposure to various cardiovascular risk factors [14,15] and WMH are associated with increased risk for future stroke [7–9]. For these reasons, some clinicians prescribe an antiplatelet agent for WMH. However, there have been no studies on the effect of antiplatelet treatment on the progression of WMH.

To the best of our knowledge, this is the first longitudinal study to assess the effects of an antiplatelet agent on the progression of WMH. A possibility of treatment selection bias was the main problem with our study design, because we could not randomly assign patients to the NAG and AG. In order to minimize potential bias, we used IPW, which is a widely used statistical technique to correct for selection bias.

In our study, there was no significant protective effect of antiplatelet agents on WMH progression. This finding might be related to the underlying mechanism leading to WMH development. The key effect of antiplatelet agents is an anti-thrombotic function by inhibiting platelet activation and aggregation. However, the underlying pathophysiological mechanism of WMH is chronic hypoperfusion mainly due to lipohyalinosis rather than atheroma formation by platelet activation [16]. Therefore, antiplatelet agents might have a relatively minimal effect on WMH progression. However, our negative finding does not negate the utility of antiplatelet treatment for patients who have atrial fibrillation or any history of ischemic stroke or systemic thromboembolism.

There were some limitations in this study that should be noted. First, we did not have detailed information on the level of risk factor control. The heterogeneity of prescribed antiplatelet medications is also a limitation of our study. Our study population was fairly young with a relatively low prevalence of vascular risk factors. There is a possibility that the results might be different in an older sample of patients who have a greater vascular risk burden. Finally, this was a single center study examining a small cohort of patients. Moreover, only Koreans were included in this study, which could limit the generalizability of our results because WMH could be associated with dietary [17] and genetic factors [18,19] that vary based on race, ethnicity and nationality. A multicenter, multinational randomized controlled study will be required to validate our result.

## Supporting information

S1 File Data set. (PDF)

## References

[1] Hachinski VC, Potter P, Merskey H. Leuko-araiosis. Arch Neurol. 1987;44: 21–23. 3800716 10.1001/archneur.1987.00520130013009

[2] Ylikoski A, Erkinjuntti T, Raininko R, Sarna S, Sulkava R, Tilvis R. White matter hyperintensities on MRI in the neurologically nondiseased elderly Analysis of cohorts of consecutive subjects aged 55 to 85 years living at home. Stroke. 1995;26: 1171–1177. 7604409 10.1161/01.str.26.7.1171

[3] Longstreth W, Manolio TA, Arnold A, Burke GL, Bryan N, Jungreis CA, et al Clinical correlates of white matter findings on cranial magnetic resonance imaging of 3301 elderly people The Cardiovascular Health Study. Stroke. 1996;27: 1274–1282. 8711786 10.1161/01.str.27.8.1274

[4] Garde E, Mortensen EL, Krabbe K, Rostrup E, Larsson H. Relation between age-related decline in intelligence and cerebral white-matter hyperintensities in healthy octogenarians: a longitudinal study. Lancet. 2000;356: 628–634. doi: 10.1016/S0140-6736(00)02604-0 10968435 10.1016/S0140-6736(00)02604-0

[5] De Leeuw F, De Groot J, Achten E, Oudkerk M, Ramos L, Heijboer R, et al Prevalence of cerebral white matter lesions in elderly people: a population based magnetic resonance imaging study. The Rotterdam Scan Study. Journal of Neurology, Neurosurgery & Psychiatry. 2001;70: 9–14. 10.1136/jnnp.70.1.9 PMC1763449 11118240

[6] Launer LJ. Epidemiology of white matter lesions. Top Magn Reson Imaging. 2004;15: 365–367. 16041288 10.1097/01.rmr.0000168216.98338.8d

[7] Vermeer SE, Hollander M, van Dijk EJ, Hofman A, Koudstaal PJ, Breteler MM. Silent brain infarcts and white matter lesions increase stroke risk in the general population the rotterdam scan study. Stroke. 2003;34: 1126–1129. doi: 10.1161/01.STR.0000068408.82115.D2 12690219 10.1161/01.STR.0000068408.82115.D2

[8] Inzitari D. Leukoaraiosis An Independent Risk Factor for Stroke? Stroke. 2003;34: 2067–2071. doi: 10.1161/01.STR.0000080934.68280.82 12829859 10.1161/01.STR.0000080934.68280.82

[9] Kuller LH, Longstreth W, Arnold AM, Bernick C, Bryan RN, Beauchamp NJ, et al White matter hyperintensity on cranial magnetic resonance imaging a predictor of stroke. Stroke. 2004;35: 1821–1825. doi: 10.1161/01.STR.0000132193.35955.69 15178824 10.1161/01.STR.0000132193.35955.69

[10] Jeon S, Yoon U, Park JS, Seo SW, Kim JH, Kim ST, et al Fully automated pipeline for quantification and localization of white matter hyperintensity in brain magnetic resonance image. International Journal of Imaging Systems and Technology. 2011;21: 193–200.

[11] Zhang Y, Brady M, Smith S. Segmentation of brain MR images through a hidden Markov random field model and the expectation-maximization algorithm. IEEE transactions on medical imaging. 2001;20: 45–57. doi: 10.1109/42.906424 11293691 10.1109/42.906424

[12] Fazekas F, Chawluk JB, Alavi A, Hurtig HI, Zimmerman RA. MR signal abnormalities at 1.5 T in Alzheimer's dementia and normal aging. American journal of roentgenology. 1987;149: 351–356. doi: 10.2214/ajr.149.2.351 3496763 10.2214/ajr.149.2.351

[13] Enzinger C, Fazekas F, Ropele S, Schmidt R. Progression of cerebral white matter lesions—clinical and radiological considerations. Journal of the neurological sciences. 2007;257: 5–10. doi: 10.1016/j.jns.2007.01.018 17321549 10.1016/j.jns.2007.01.018

[14] Allan CL, Zsoldos E, Filippini N, Sexton CE, Topiwala A, Valkanova V, et al Lifetime hypertension as a predictor of brain structure in older adults: cohort study with a 28-year follow-up. The British Journal of Psychiatry. 2015;206: 308–315. doi: 10.1192/bjp.bp.114.153536 25497301 10.1192/bjp.bp.114.153536 PMC4381190

[15] Swan GE, DeCarli C, Miller B, Reed T, Wolf P, Jack L, et al Association of midlife blood pressure to late-life cognitive decline and brain morphology. Neurology. 1998;51: 986–993. 9781518 10.1212/wnl.51.4.986

[16] Hatazawa J, Shimosegawa E, Satoh T, Toyoshima H, Okudera T. Subcortical hypoperfusion associated with asymptomatic white matter lesions on magnetic resonance imaging. Stroke. 1997;28: 1944–1947. 9341700 10.1161/01.str.28.10.1944

[17] Gardener H, Scarmeas N, Gu Y, Boden-Albala B, Elkind MS, Sacco RL, et al Mediterranean diet and white matter hyperintensity volume in the Northern Manhattan Study. Archives of neurology. 2012;69: 251–256. doi: 10.1001/archneurol.2011.548 22332193 10.1001/archneurol.2011.548 PMC3281550

[18] Tran T, Cotlarciuc I, Yadav S, Hasan N, Bentley P, Levi C, et al Candidate-gene analysis of white matter hyperintensities on neuroimaging. Journal of Neurology, Neurosurgery & Psychiatry. 2016;87: 260–266. 10.1136/jnnp-2014-309685 PMC4789815 25835038

[19] Choi JC. Genetics of cerebral small vessel disease. J Stroke. 2015;17: 7–16. doi: 10.5853/jos.2015.17.1.7 25692103 10.5853/jos.2015.17.1.7 PMC4325630

