# Supplementary material for: Is antiplatelet treatment effective at attenuating the progression of white matter hyperintensities?
Source: PLoS One. 2017 Apr 20;12(4):e0176300. doi: 10.1371/journal.pone.0176300 (PMC5398663; doi:10.1371/journal.pone.0176300)
Supplement: S1 File — (PDF) [file pone.0176300.s001.pdf]

No	Age	Sex	Group	HTN	DM	Lipid	Sm	Base DWM	Base PVWM	Base Total	F/U DWM	F/U PVWM	F/U Total	MRI interval (days)
1	66	Female	AG	No	No	No	No	2751.49	3755.53	6507.02	4662.99	5066.22	9729.21	1765
2	51	Female	AG	No	No	No	No	638.77	450.26	1089.03	651.8	654	1305.8	2159
3	50	Male	NAG	No	No	No	No	243.53	371.7	615.23	294.69	1725.49	2020.18	1730
4	52	Female	AG	Yes	No	No	No	14976.24	14457.93	29434.17	20366.71	46060.85	66427.6	1922
5	68	Female	AG	No	No	No	No	5787.73	1438.5	7226.23	6091.61	4967.12	11058.7	1880
6	66	Male	AG	Yes	No	No	No	232.64	2067.87	2300.51	333.26	2140.53	2473.79	1719
7	57	Female	AG	No	No	No	No	243.53	986.93	1230.46	909.93	2737.55	3647.48	1982
8	68	Male	AG	Yes	Yes	No	No	906.6	2967.83	3874.43	2714.05	3489.5	6203.55	1733
9	57	Female	AG	No	No	Yes	No	261.08	1546.61	1807.69	444.33	1844.39	2288.72	1692
10	72	Female	AG	No	No	No	No	589.34	2871.08	3460.42	1452.63	3396.48	4849.11	2094
11	52	Female	AG	Yes	No	No	No	111.08	2473.71	2584.79	458.84	3139.4	3598.24	1869
12	63	Female	NAG	No	No	No	No	138.15	869.16	1007.31	299.58	884.01	1183.59	2051
13	61	Male	AG	No	No	No	No	2809.62	863.84	3673.46	3908.26	1034.59	4942.85	1888
14	62	Female	AG	No	No	No	No	149.53	1773.04	1922.57	158.98	1856.16	2015.14	1763
15	70	Female	NAG	Yes	Yes	No	No	203.19	468.83	672.02	350.27	536.39	886.66	1741
16	62	Male	AG	Yes	No	No	No	1597.61	5502.36	7099.97	4476.9	16749.61	21226.5	1890
17	55	Male	NAG	No	No	No	Yes	896.19	1483.79	2379.98	1406.16	1809.4	3215.56	2044
18	50	Male	AG	No	No	No	No	718.44	1599.38	2317.82	1560.42	1702.98	3263.4	1729
19	58	Male	NAG	No	No	No	Yes	575.36	1391.17	1966.53	610.2	1483.79	2093.99	1824
20	88	Female	NAG	No	No	No	No	171.34	565.41	736.75	175.43	600.97	776.4	1777
21	57	Female	NAG	No	No	Yes	No	1013.26	2006.83	3020.09	1144.92	2719.26	3864.18	1854
22	56	Female	NAG	No	No	No	No	171.34	1884.15	2055.49	780.63	1978.86	2759.49	1821
23	68	Female	NAG	No	No	No	No	427.25	1298.85	1726.1	1245.96	2583.68	3829.64	1757
24	53	Female	NAG	No	No	No	No	31.91	317.32	349.23	62.04	449.6	511.64	1860
25	53	Female	AG	No	No	No	No	384.53	495.61	880.14	396.45	1075.3	1471.75	1756
26	60	Female	AG	No	No	No	No	2035.68	1408.82	3444.5	4512.46	3974.86	8487.32	2038
27	68	Female	AG	No	No	No	No	4567.3	3994.79	8562.09	4588.84	4443.55	9032.39	1637
28	57	Female	AG	Yes	No	No	No	346.37	2004.59	2350.96	826.68	3406.87	4233.55	1795

29	52	Female	AG	No	No	No	No	29.91	264.89	294.8	155.09	496.29	651.38	1951
30	61	Female	AG	Yes	No	No	No	3081.71	1615.65	4697.36	5919.2	2817.44	8736.64	2028
31	63	Male	NAG	No	No	No	No	1966.11	7509.7	9475.81	7026.06	7919.18	14945.2	2019
32	53	Male	AG	Yes	Yes	Yes	No	145.8	969.12	1114.92	218.43	1468.26	1686.69	1925
33	51	Female	NAG	No	No	No	No	51.27	0	51.27	117.14	0	117.14	1818
34	56	Female	NAG	No	No	No	No	89.72	620.39	710.11	100.81	881.84	982.65	1740
35	55	Female	NAG	No	No	No	No	1793.87	4887.31	6681.18	2034.25	6296.62	8330.87	1952
36	59	Female	AG	No	No	No	No	12074.59	7052.42	19127.01	22963.32	9925.61	32888.9	2022
37	54	Male	AG	Yes	No	No	No	128.18	316.17	444.35	413.6	567.41	981.01	1751
38	57	Female	AG	Yes	Yes	Yes	No	662.84	1654.96	2317.8	1171.9	1987.48	3159.38	2210
39	64	Female	AG	No	No	No	No	158.08	328.98	487.06	289.85	525.13	814.98	1761
40	52	Male	AG	Yes	Yes	No	Yes	196.54	534.06	730.6	289.17	679.33	968.5	1763
41	56	Male	AG	No	No	No	No	367.43	1867.05	2234.48	448.64	1908.63	2357.27	2050
42	76	Female	AG	No	No	No	No	2144.81	3877.65	6022.46	2708.87	4363.14	7072.01	1763
43	60	Male	AG	Yes	No	No	No	414.84	2626.17	3041.01	723.75	2984.31	3708.06	2112
44	47	Female	NAG	No	No	No	No	115.54	350.91	466.45	542.82	542.82	1085.64	1741
45	62	Female	AG	Yes	No	Yes	No	153.81	657.96	811.77	473.05	753.52	1226.57	2017
46	58	Female	AG	Yes	No	Yes	No	427.63	880.93	1308.56	651.39	1245.91	1897.3	2140
47	59	Male	AG	No	No	No	Yes	289.17	1661.17	1950.34	816.8	3070.75	3887.55	1812
48	53	Female	NAG	No	No	No	No	418.64	2542.06	2960.7	434.9	3158.98	3593.88	1961
49	68	Male	NAG	No	No	No	No	243.53	337.53	581.06	351.56	1523.85	1875.41	1780
50	63	Female	NAG	Yes	Yes	No	No	325.36	1320.73	1646.09	425.89	1587.12	2013.01	1758
51	61	Female	AG	No	No	No	No	465.7	679.33	1145.03	565.44	726.67	1292.11	1749
52	64	Female	NAG	Yes	Yes	No	No	0	2371.18	2371.18	2155.86	2648.3	4804.16	1974
53	62	Male	AG	No	No	No	No	205.83	1213.54	1419.37	303.73	2188.18	2491.91	2137
54	60	Female	NAG	No	No	No	No	1482.8	1229.95	2712.75	1761.27	1666.92	3428.19	2326
55	51	Male	AG	No	No	No	No	68.36	0	68.36	98.23	0	98.23	1777
56	62	Female	NAG	No	No	No	No	256.36	367.45	623.81	1523.85	2727.16	4251.01	2029
57	58	Male	AG	Yes	Yes	No	Yes	1726.17	3478.04	5204.21	1909.92	3636.31	5546.23	2093

58	66	Male	NAG	Yes	No	Yes	No	987.4	2994.98	3982.38	1516.72	4368.35	5885.07	2089
59	54	Male	NAG	Yes	No	No	No	646.39	598.14	1244.53	670.77	708.96	1379.73	2124
60	63	Male	AG	No	No	No	No	1184.54	4105.8	5290.34	1491.08	5002.72	6493.8	1877
61	64	Male	NAG	No	No	No	No	538.33	837.41	1375.74	942.03	985.25	1927.28	1790
62	67	Female	NAG	No	No	No	No	666.49	1332.97	1999.46	970.77	1482.8	2453.57	2274
63	50	Male	AG	No	No	No	No	7610.12	4666.06	12276.18	11538.61	11936.67	23475.3	2099
64	67	Female	NAG	No	No	No	No	1431.28	3699.97	5131.25	4999.08	6498.29	11497.4	1403
65	61	Female	NAG	No	No	No	No	511.8	1846.46	2358.26	525.73	2243.67	2769.4	2146
66	68	Female	NAG	No	No	No	No	30.02	497.42	527.44	45.14	513.45	558.59	1773
67	55	Female	AG	No	No	No	No	91.77	990.19	1081.96	102.33	1138.41	1240.74	1785
68	64	Female	NAG	No	No	No	No	448.61	1691.89	2140.5	621.81	2362.52	2984.33	2005
69	64	Female	AG	Yes	No	No	No	1174.93	1038.22	2213.15	10201.63	4810.65	15012.3	1791
70	53	Female	NAG	No	No	No	No	166.62	0	166.62	284.64	0	284.64	1788
71	61	Male	AG	No	No	No	No	139.58	1426.85	1566.43	432.61	1507.72	1940.33	1888
72	63	Female	AG	No	No	No	No	427.24	1465.44	1892.68	1314.47	1572.97	2887.44	2117
73	59	Female	AG	Yes	No	Yes	No	902.32	1064.82	1967.14	953.17	1139.98	2093.15	2128
74	69	Female	AG	No	No	No	No	210.93	1915.21	2126.14	2834.46	5173.89	8008.35	1769
75	67	Female	AG	No	No	No	No	166.73	1057.25	1223.98	666.49	1332.97	1999.46	2076
76	58	Female	NAG	No	No	No	No	287.68	953.2	1240.88	418.75	1385.5	1804.25	2088
77	62	Male	AG	Yes	Yes	No	No	206.79	661.74	868.53	222.73	1319.26	1541.99	1917
78	58	Female	NAG	No	No	No	No	81.18	739.14	820.32	459.76	1964.44	2424.2	1788
79	77	Female	AG	No	No	No	No	76.9	1174.93	1251.83	129.08	1519.88	1648.96	1777
80	55	Female	AG	No	No	No	No	149.67	542.03	691.7	155.09	543.1	698.19	1762
81	65	Male	NAG	No	No	No	No	21.36	0	21.36	62.04	211.96	274	1786
82	84	Female	NAG	No	No	No	No	86.15	4001.82	4087.97	713.43	4379.11	5092.54	1757
83	57	Male	AG	No	No	No	No	1679.78	970.25	2650.03	1743.59	1611.76	3355.35	1792
84	64	Female	AG	No	No	No	No	261.57	966.12	1227.69	270	994.83	1264.83	2180
85	66	Female	AG	No	No	No	No	645.56	1584.18	2229.74	1121.15	2438.45	3559.6	1804
86	68	Male	AG	Yes	Yes	No	No	2247.28	13560.6	15807.88	6937.69	22239.88	29177.6	2110

87	69	Male	NAG	No	No	No	No	697.04	2026.98	2724.02	754.29	2046.71	2801	1792
88	69	Male	AG	Yes	Yes	No	No	321.26	843.83	1165.09	1553.58	2237.31	3790.89	1780
89	50	Female	NAG	Yes	No	No	No	346.37	1520.57	1866.94	462.6	3406.87	3869.47	1860
90	59	Male	AG	Yes	Yes	No	No	2031.71	4487.35	6519.06	6787.96	19790.04	26578	1774
91	63	Female	NAG	No	No	No	No	188.07	315.35	503.42	227.46	367.59	595.05	2267
92	51	Male	NAG	No	No	No	No	189.54	1412.91	1602.45	392.89	3618.71	4011.6	1921
93	51	Female	NAG	No	No	No	No	155.09	144.75	299.84	253.32	268.82	522.14	1926
